# Chronic intermittent hypoxia, a hallmark of obstructive sleep apnea, promotes 4T1 breast cancer development through endothelin-1 receptors

**DOI:** 10.1038/s41598-022-15541-8

**Published:** 2022-07-28

**Authors:** Mélanie Minoves, Sylvain Kotzki, Florence Hazane-Puch, Emeline Lemarié, Sophie Bouyon, Julien Vollaire, Brigitte Gonthier, Jean-Louis Pépin, Véronique Josserand, Anne Briançon-Marjollet, Diane Godin-Ribuot

**Affiliations:** 1 HP2 Laboratory, INSERM U1030, Grenoble Alpes University, 38000 Grenoble, France; 2 Grenoble Alpes University Hospital, 38000 Grenoble, France; 3 Institute for Advanced Biosciences, INSERM U1209 / CNRS UMR5309, Grenoble Alpes University, 38000 Grenoble, France

**Keywords:** Breast cancer, Cell migration, Mechanisms of disease, Sleep disorders

## Abstract

The association between obstructive sleep apnea (OSA) and cancer is still debated and data are scarce regarding the link between OSA and breast cancer progression. Since conclusive epidemiological studies require large sample sizes and sufficient duration of exposure before incident cancer occurrence, basic science studies represent the most promising approach to appropriately address the topic. Here we assessed the impact of intermittent hypoxia (IH), the major hallmark of OSA, on the development of breast cancer and explored the specific involvement of the endothelin signaling pathway. Original in vitro and in vivo models were used where 3D-spheroids or cultures of murine 4T1 breast cancer cells were submitted to IH cycles, and nude NMRI mice, orthotopically implanted with 4T1 cells, were submitted to chronic IH exposure before and after implantation. The role of the endothelin-1 in promoting cancer cell development was investigated using the dual endothelin receptor antagonist, macitentan. In vitro exposure to IH significantly increased 4T1 cell proliferation and migration. Meta-analysis of 4 independent in vivo experiments showed that chronic IH exposure promoted tumor growth, assessed by caliper measurement (overall standardized mean difference: 1.00 [0.45–1.55], p < 0.001), bioluminescence imaging (1.65 [0.59–2.71]; p < 0.01) and tumor weight (0.86 [0.31–1.41], p < 0.01), and enhanced metastatic pulmonary expansion (0.77 [0.12–1.42]; p = 0.01). Both in vitro and in vivo tumor-promoting effects of IH were reversed by macitentan. Overall, these findings demonstrate that chronic intermittent hypoxia exposure promotes breast cancer growth and malignancy and that dual endothelin receptor blockade prevents intermittent hypoxia-induced tumor development.

## Introduction

Obstructive sleep apnea (OSA) is a common chronic respiratory disorder estimated to affect almost 1 billion people world-wide^1^. It is characterized by nocturnal repetitive upper airway obstructions resulting in repetitive hypoxia/reoxygenation sequences leading to intermittent hypoxia (IH). OSA is well known to promote neurocognitive impairment and cardiometabolic complications^2,3^ and an increasing number of studies have explored its role as a potential new risk factor for cancer. Hence, Nieto et al. were the first to show an association between OSA severity and cancer mortality in the Wisconsin Sleep Cohort^4^. A decade later, two recent multicenter retrospective clinical cohort studies have reported an association between overnight hypoxia severity and an increased cancer incidence in OSA patients^5,6^.

According to the Globocan 2020 data of the World Health Organization, breast cancer in women represents an estimated 2.3 million new cases (11.7%) thus outperforming lung cancer as the most commonly diagnosed cancer worldwide^7^. Since breast cancer prognosis is highly dependent on early diagnosis, identification and monitoring of risk factors for breast cancer are thus highly important in order to improve the management of the disease. An increasing number of studies have explored the potential role of OSA as a new risk factor for breast cancer. Indeed two studies in women with sleep disordered breathing and a specific investigation in patients with OSA have found an increased risk of breast cancer^8–10^. These results have been strengthened by Yap et al. in a recent meta-analysis of 7 studies comprising a cohort of 5,370,466 patients which showed that patients suffering from OSA had a 36% increase in breast cancer risk, with a significantly larger association after at least five years of follow-up^11^).

One of the potential mechanisms linking OSA and cancer is chronic nocturnal IH which has been shown to play a major role in multiple deleterious consequences of OSA by inducing oxidative stress-related mechanisms, such as expression of the hypoxia-inducible factor 1 (HIF-1) transcription factor and of its deleterious target genes^12,13^. Notably, the endothelin-1 (ET-1) gene has been shown to be overexpressed in OSA patients^14–16^ and in animals exposed to IH^12,17,18^ where it promotes cardiovascular^17,19,20^ and metabolic^21^ alterations.

The hypothesis associating OSA, IH and cancer is based on the well-described link between hypoxia and cancer. Indeed, sustained hypoxic conditions in the tumor microenvironnement activate HIF-1 downstream pathways^22,23^ and notably the endothelin axis^24^ which has been shown to promote angiogenesis, cell proliferation and migration, metastasis and chemoresistance^25,26^. In OSA patients, systemic intermittent hypoxia may add to tumor hypoxia to enhance these effects. Indeed, experimental studies have demonstrated that IH per se has the ability to induce angiogenesis through endothelial cell migration mediated by the HIF-1 pathway^27,28^. Moreover, Almendros et al. have shown that IH-exposed mice exhibited larger melanoma tumors^29^ and enhanced lung metastasis^30^ compared to controls. The effects of IH on melanoma lung metastasis were confirmed by Li et al. along with an involvement of oxidative stress and downstream HIF-1 and nuclear factor-kappa B pathways^31^.

Regarding breast cancer, no preclinical data have been able so far to objectively demonstrate a link between chronic IH exposure, similar to that encountered in OSA, and breast cancer development. The present study was thus performed based on the hypothesis that female OSA patients might have an increased risk of developing breast cancer through synergetic mechanisms between the local tumor-linked hypoxia and the systemic OSA-related intermittent hypoxia thus potentiating the activation of tumor-promoting signaling pathways.

For this, we used a variety of technical approaches to investigate the effects of IH on in vitro and in vivo models of breast cancer growth and invasiveness. In view of the powerful efficacy of endothelin receptor blockade in preventing the cardiometabolic alterations induced by OSA-related chronic IH exposure, we also investigated the effects of macitentan, a dual inhibitor of endothelin receptors, on tumor initiation and development.

## Materials and methods

### Drugs and chemicals

Macitentan was graciously provided by Actelion Pharmaceuticals, Allschwil, Switzerland.RPMI-1640 medium, rat tail type I collagen, methycellulose Methocell®MC were obtained from Sigma Aldrich (Saint-Quentin-Fallavier, France). CellTiter96® MTT (3-(4,5-dimethylthiazol-2-yl)-2,5-diphenyltetrazolium bromide) and D-luciferin were purchased from Promega France (Charbonnières-les-Bains, France). Inactivated Fetal Bovine Serum Gibco was obtained from Thermofisher Waltham (Massachusetts, USA). Epidermal growth factor, basic fibroblast growth factor and NeuroBrew-21 were all bought from Miltenyi Biotec (Bergisch Gladbach, Germany). Endothelin-1 ELISA kit was provided by Enzo Life Sciences (Villeurbanne France) and Isorane® by Axience (Pantin, France).

### Murine breast cancer cells

Murine 4T1 breast cancer cells were selected to model stage IV human breast cancer with pulmonary metastatic dissemination. 4T1rvluc2 cells (provided by the OPTIMAL platform, Grenoble, France), stably transfected with the firefly luciferase gene in order to perform in vivo bioluminescence imaging, were used in all experiments and will be referred to as 4T1 in the manuscript. They were maintained in in RPMI-1640 medium containing 10% FCS and antibiotics.

### In vitro intermittent hypoxia exposure

Cultured 4T1 cells were exposed to IH cycles alternating 5 min of normoxia (16% PO_2_) and 5 min of hypoxia (2% PO_2_) or to similar normoxia (N) cycles for control experiments, as previously described^32^. Briefly, this system is adapted from commercial gas-permeable dishes (Zell-Kontakt, Germany) and uses custom-made plate holders (SMTEC, Nyon, Switzerland) connected to gas blenders (Gas Blender 100, MCQ Instruments, Rome, Italy) and hosted in a standard cell culture incubator (SANYO, MCO-15AC). The small volume of air in the holders is rapidly renewed, thus allowing fast cycling.

This system induced oxygen pressure cycles in culture medium ranging from 25 to 120 mmHg during IH^32^. In accordance with the apnea pattern of OSA patients, IH cycles were applied for 8 h during the animals sleep period followed by 16 h of normoxia. This was repeated daily for up to 4 days according to the experiments.

### Wound healing assays

4T1 cells were seeded (7.5 × 10^3^/well) in 24-well semipermeable plates (Zell-Kontakt, Nörten-Hardenberg, Germany) pre-coated with type I collagen and allowed to reach 90% confluence. One day after seeding, wound healings were performed by scratching the wells from top to bottom with a sterile 100-μl cone. Cells were then incubated in medium supplemented or not with 5 µM macitentan and exposed to N or IH for 4 days. Wound repair was assessed daily during 5 consecutive days using a camera-coupled optical microscope (× 10 magnification; Olympus CK2; Olympus, Norfolk, USA). Repaired areas were determined by subtracting cell-free areas at various timepoints from initial cell-free areas (ImageJ software, NIH, Bethesda, USA). Values of filled areas were normalized relative to control areas.

### Proliferation assays

For proliferation assays, 4T1 cells (3 × 10^3^) were seeded in 96-well semipermeable plates pre-coated with type I collagen, in medium with or without 5 µM macitentan, and submitted to a 2-day N or IH exposure. Cell viability was then assessed using MTT (3-(4,5-dimethylthiazol-2-yl)-2,5-diphenyltetrazolium bromide) staining.

### Transwell migration assays

4T1 cells (10^6^) were seeded in the top chamber of Transwell 8.0 μm pore polycarbonate membrane polycarbonate membrane cell culture inserts (Corning®, CLS3422-48EA, Sigma Aldrich, USA) coated with type I collagen, in serum-free RPMI-1640 medium supplemented or not with 5 µM macitentan. 4T1 cells were loaded in the upper well of each chamber. The bottom chamber was filled with 600 µl of complete RPMI-1640 medium with 10% FBS as chemoattractant. After 2 days of N or IH exposure, the medium was aspirated and cells on the top chamber were gently removed using a cotton swab. Cells attached to the lower layer of the membrane were then fixed with absolute ethanol, stained with 0.2% crystal violet for 10 min at room temperature and imaged using a camera-coupled optical microscope (× 10 magnification; Olympus CK2, Olympus, USA). Migration ability was estimated by OD measurement (595 nm) after solubilization in acetic acid.

### Spheroid proliferation and invasiveness

4T1rvluc2 cells were cultured in uncoated 96-well tissue plates with U-shaped bottoms (Greiner Bio-One, Frickenhausen, Germany) to allow the formation of one spheroid/well. Two thousand cells/well were loaded in medium enriched with 10% methylcellulose and the plates were centrifuged at 500×*g* for 5 min to initiate the process of spheroid formation. Three days later, spheroid formation was assessed by microscopic visualization. Spheroids were isolated and placed in 24-well semipermeable bottom plates pre-coated with type I collagen along with 1 ml of serum-free medium. Serum was replaced by adding 20 ng/mL of epidermal growth factor, 20 ng/mL of basic fibroblast growth factor and 1X NeuroBrew-21.

Spheroid diameter was measured using a graduated scale placed into the objective of a camera-coupled optical microscope and pictures were taken twice daily (× 10 magnification Axiovert 25; Carl Zeiss, Hombrechtikon, Switzerland).

### Measurement of ET-1 levels in culture media

ET-1 levels in culture media of 4T1rvluc2 cells were assessed using ELISA kit according to the supplier’s recommendations.

### Murine orthotopic breast cancer model

Animal experiments were performed according to the Declaration of Helsinki conventions for the Use and Care of Animals and approved by the Institutional Ethics Committee for Animal Research on August 28th 2018 (agreement No. 2016012818193391). The study was conducted on 5-week-old female NMRI nude mice (Janvier Labs, Le Genest-Saint-Isle, France) weighing 20 to 25 g. Animals were housed in controlled light and temperature conditions with food and tap water ad libitum and were acclimated for 1 week prior to the beginning of the experiments.

### In vivo intermittent hypoxia exposure

**Figure 1:**
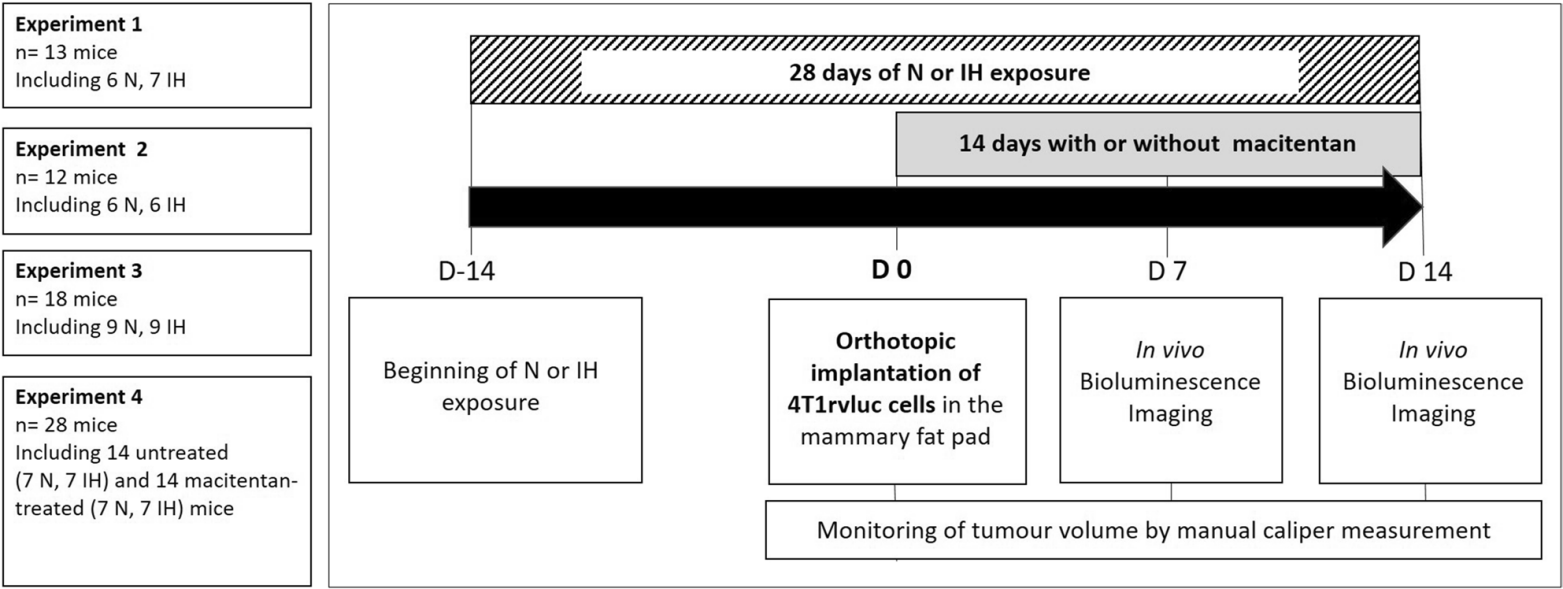
Four independent experiments were conducted. Mice were divided into normoxic and hypoxic groups for experiments 1, 2 and 3. In experiment 4, mice were splittd into 4 groups to assess the both effects of exposure to IH and macitentan. For all groups (n = 71 mice), the experiment consists of a 28-day exposure to HI or normoxia with implantation of 4T1rvluc tumor cells after fourteen days (D0). Quantification of tumor growth by bioluminescence imaging is performed at 7 days and 14 days post-implantation in all groups. In experiment 4, mice received treatment (macitentan or control) every day from implantation. Regular follow-up of tumour volume was performed by caliper measurement.

Four independent experiments were performed with different numbers of mice as summarized in Fig. 1. Altogether seventy-one female NMRI nude mice were used in this study and were divided in N (n = 35) and IH (n = 36) groups.

Evaluation of the effects of intermittent hypoxia was repeated 4 times through 4 independent experiments. The effect of macitentan treatment was evaluated in experiment 4. Mice were exposed daily during 28 days: 14 days before (D-14) and 14 days after (D 14) tumor implantation (D0). Intermittent hypoxia (IH) exposure consisted of 8 consecutive hours of 1-min IH cycles, comprising 30 s at 21% FiO_2_ and 30 s at 5% FiO_2_, or to similar air-air cycles for normoxic (N) exposure. After 4T1 cell implantation at D0, the animals were treated or not with oral macitentan throughout the remaining 14 days of N or IH exposure.

As previously described^8^, mice were exposed daily to 8 consecutive hours of 1-min IH cycles (30 s at 21% FiO_2_ and 30 s at 5% FiO_2_) or to similar air-air cycles. FiO2 level in housing cages was monitored throughout the experiments (ML206 gas analyzer; ADInstruments, UK). After 14 days of N or IH exposure, mice were injected with 5 × 10^5^ 4T1 cells into the mammary fat pad^33^ and then re-exposed for 14 additional days before primary tumor resection. Fourteen of the mice were submitted to the same N (n = 7) or IH (n = 7) protocol but were treated daily, following tumor implantation, with 30 mg/kg macitentan added to their food. Tumor growth (volume = 0.5 × length × width^2^) was monitored thrice a week using an electronic caliper.

### Bioluminescence imaging

Proliferation and thoracic dissemination of 4T1 cells were quantified by in vivo bioluminescence imaging (IVIS Kinetic, PerkinElmer, Villebon-sur-Yvette, France) on days 7 and 14 following implantation. Five minutes before imaging, vigil mice received an intraperitoneal injection of 150 µg/g of d-luciferin. They were then anesthetized (isoflurane 4% for induction and 1.5% thereafter) and placed in the BLI system (IVIS Kinetic, PerkinElmer, Villebon-sur-Yvette, France), in supine position.

Signal quantifications were carried out by drawing regions of interest on the primary tumor and thorax areas (LivingImage software; PerkinElmer, Villebon-sur-Yvette, France). Results were expressed as photons per second (ph/s).

### Statistical analysis

Comparison between two groups were performed by t-test or Mann–Whitney test, while comparisons of several groups were performed by two-way ANOVA or Kruskal–wallis test followed by the appropriate post hoc tests. Time-series effects were analyzed by repeated-measures analyses of variance. The choice of parametric or nonparametric tests depended on the normal distribution of values and on the equality of variances. Frequencies were analyzed by Chi-squared tests. In cell culture experiments, to take into account the fact that several duplicate wells were usually done in a single independent experiment, the experiment was included as a random factor. A two-tailed p-value < 0.05 was considered significant. Continuous data were represented as mean ± standard error of mean or median ± 10%–90% range, depending on the normality of values.

A meta-analytical method was performed on repeated independent in vivo experiments to evaluate the global Hedge’s g effect size (see supplementary methods for details)^34^. By convention, overall effect sizes of 0.2, 0.5 and 0.8 standard deviations are considered as “small”, “medium” and “large”, respectively.

Statistical analysis and meta-analysis were performed using SPSS Statistics (version 20.0, IBM, Armonk, USA) and RStudio (version 1.2.1335, RStudio, Boston, USA) software, respectively.

### Ethics approval

Animal experiments were performed according to the Declaration of Helsinki conventions for the Use and Care of Animals and approved by the Institutional Ethics Committee for Animal Research on August 28th 2018 (agreement No. 2016012818193391).

## Results

### In vitro proliferation and migration of 4T1 breast tumor cells are increased by intermittent hypoxia exposure

**Figure 2:**
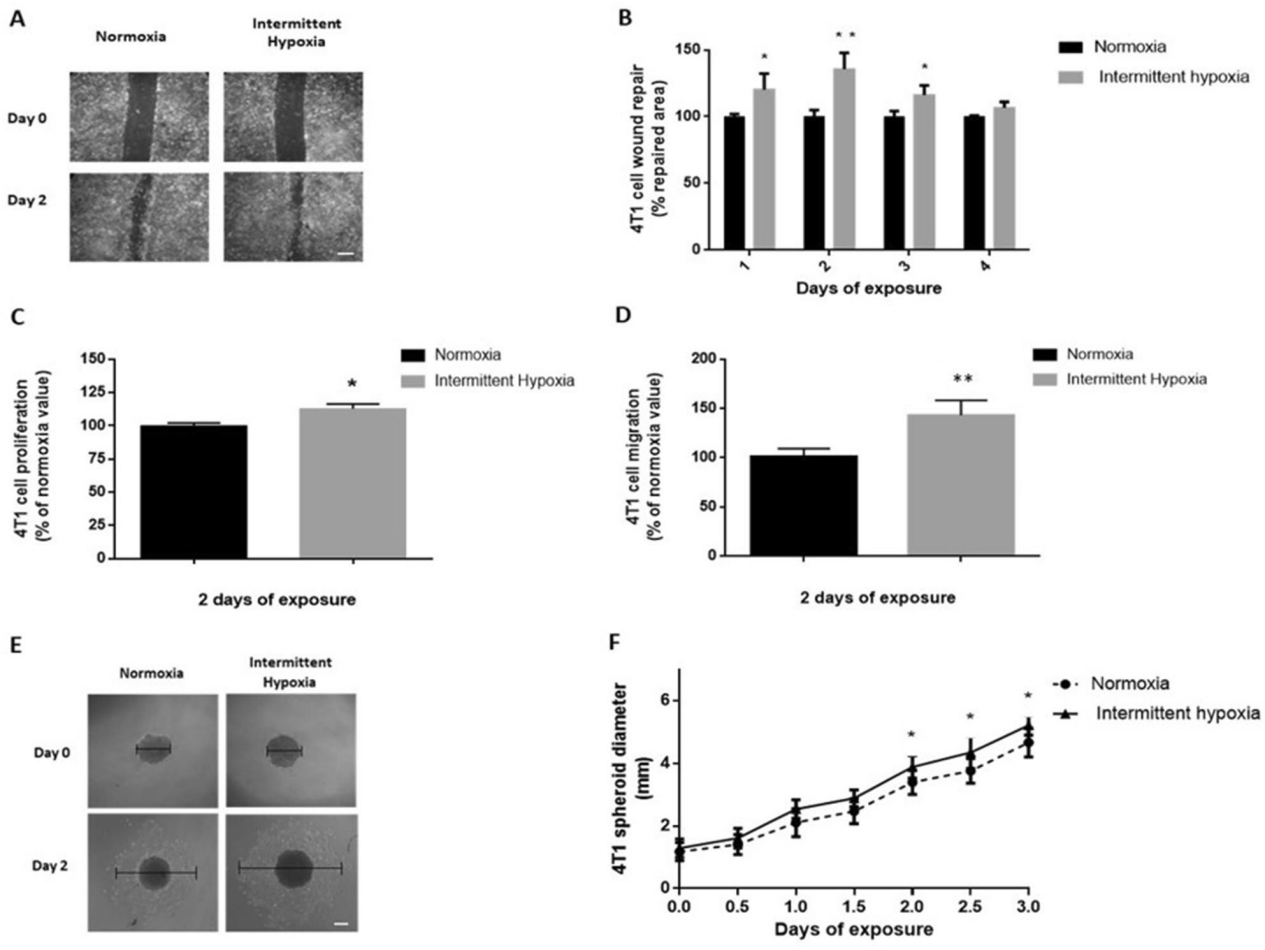
In vitro intermittent hypoxia exposure increases breast cancer cell proliferation, migration and expansion. (**A**) Representative illustrations of 4T1 cell migration, assessed by wound healing repair, before and after 2 days of normoxia or intermittent hypoxia exposure (white scale bar = 300 µm). (**B**) Wound healing, expressed as a % of normoxia values, of 4T1 cells exposed to 4 days of normoxia or intermittent hypoxia; n = 4 independent experiments/group (12 wells/group: 3 wells/experiment). *p < 0.05 and **p < 0.01, repeated measure one-way ANOVA. Data are presented as mean ± SEM. (**C**) Proliferation, expressed as a % of normoxia values, of viable 4T1 cells quantified by 3-(4,5-dimethylthiazol-2-yl)-2,5-diphenyltetrazolium bromide (MTT) staining after 2 days of normoxia or intermittent hypoxia exposure; n = 4 independent experiments/group (12 wells/group: 3 wells/experiment). *p < 0.05, Mann–Whitney test. Data are presented as median ± range. (**D**) Migration, expressed as a % of normoxia values, of 4T1 cells after 2 days of normoxia or intermittent hypoxia exposure; n = 4 independent experiment/group (8 wells/group: 2 transwells/experiments). **p < 0.01, Mann–Whitney test. Data are presented as median ± range. (**E**) Representative illustrations of 4T1 spheroid expansion before and after 2 days of normoxia or intermittent hypoxia exposure (white scale bar = 300 µm). (**F**) 4T1 spheroid expansion in response to 3 days of normoxia or intermittent hypoxia; n = 3 experiments/group (at least 18 wells/group: 6 to 12 wells/experiment). *p < 0.05, repeated measures ANOVA. Post-hoc analysis showed significant differences at days 2, 2.5 and 3 of exposure. *p < 0.05. Data are presented as mean ± SEM.

Kinetics of 2D wound healing was increased in cells exposed to IH for 4 days compared to normoxia (p < 0.05) (data not shown). Significant differences were observed on the first 3 days of IH exposure (p < 0.05 at days 1 and 3, p < 0.01 at day 2) (Fig. 2A,B). Proliferation and migration of 4T1 cells was significantly enhanced respectively by 14% and 43% following a 2-day IH exposure compared to normoxia (p < 0.05) (Fig. 2C,D).

These results were further confirmed in 3D 4T1 spheroids (Fig. 2E,F). Indeed, IH significantly enhanced spheroid expansion up to 20% compared to normoxia (p < 0.01). Post-hoc analysis showed significant differences on days 2, 2.5 and 3 of IH exposure (p < 0.05) (Fig. 2E,F).

### In vitro tumor promoting effects of intermittent hypoxia are reversed by non-selective endothelin-1 receptor antagonism

**Figure 3:**
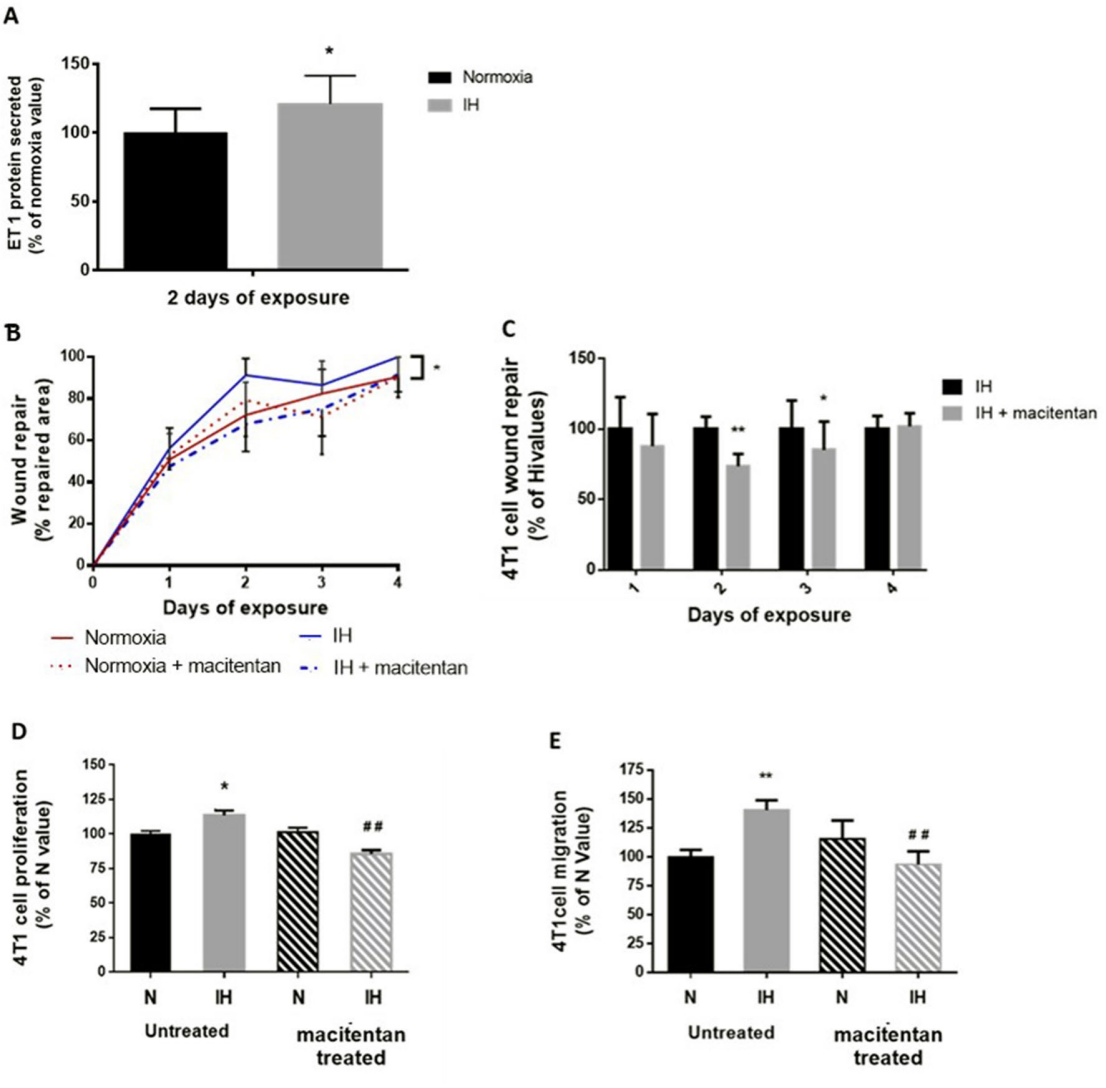
Non-selective endothelin receptor blockade prevents the increase in 4T1 cells proliferation and migration induced by in vitro intermittent hypoxia exposure. Effects of macitentan (5 µM in culture medium) on wound healing were evaluated during 4 days of normoxia (N) or intermittent hypoxia (IH) exposure and on proliferation and migration after 2 days of N or IH exposure. (**A**) 4T1 cells exposed to IH secreted more endothelin-1 (ET-1) than normoxic cells. n = 7 independent experiments/group. *p = 0.049, Mann–Whitney test. Data are presented as median ± range. (**B**) Macitentan treatment significantly reduced the wound-healing promoting effect (expressed as a % of repaired area) of IH exposure. n = 4 independent experiments/group (at least 9 wells/group: 3–4 wells/experiment). **p = 0.01 N vs. IH, repeated measures ANOVA. (**C**) Effect of macitentan on IH-induced wound healing was maximal at days 2 and 3 of exposure. n = 4 independent experiments/group (at least 9 wells/group: 3–4 wells/experiment). ***p = 0.012 and **p = 0.001, repeated measures ANOVA. (**D**) Increase in cell proliferation (expressed as a % of N values) induced by 2 days of IH exposure was significantly abolished in macitentan-treated cells. n = 3 independent experiments/group (at least 9 wells/group: 3–4 wells/experiment). *p < 0.05 vs. N, ^##^p < 0.01 vs. untreated IH, two-way ANOVA. Data are presented as mean ± SEM. (**E**) Increase in cell migration (expressed as a % of N values) induced by 2 days of IH exposure was not seen in macitentan-treated cells. n = 4 independent experiments/group (at least 2 wells/experiment). **p < 0.01 vs. N, ^##^p < 0.01 vs. untreated IH, Kruskall-Wallis test. Data are presented as median ± range.

We observed that ET-1 secretion by 4T1 cells was increased by 21% after 2 days of IH exposure (p < 0.05) (Fig. 3A). We thus used macitentan, a non-selective ET receptors antagonist, to investigate the impact of the resulting increase in ET-1 signaling. In normoxic conditions, macitentan treatment did not affect wound healing or cell migration. In contrast, macitentan significantly reduced wound healing under IH exposure (p < 0.01) (Fig. 3B), mainly after 2 (24.5% decrease; p = 0.001) and 3 (15% decrease; p < 0.01) days of exposure (Fig. 3B). In addition, macitentan significantly reduced 4T1 cell proliferation (p < 0.001) (Fig. 3D) and migration (p < 0.01) (Fig. 3E).

### Intermittent hypoxia exposure accelerates tumor growth in an orthotopic rodent model of breast cancer

**Figure 4:**
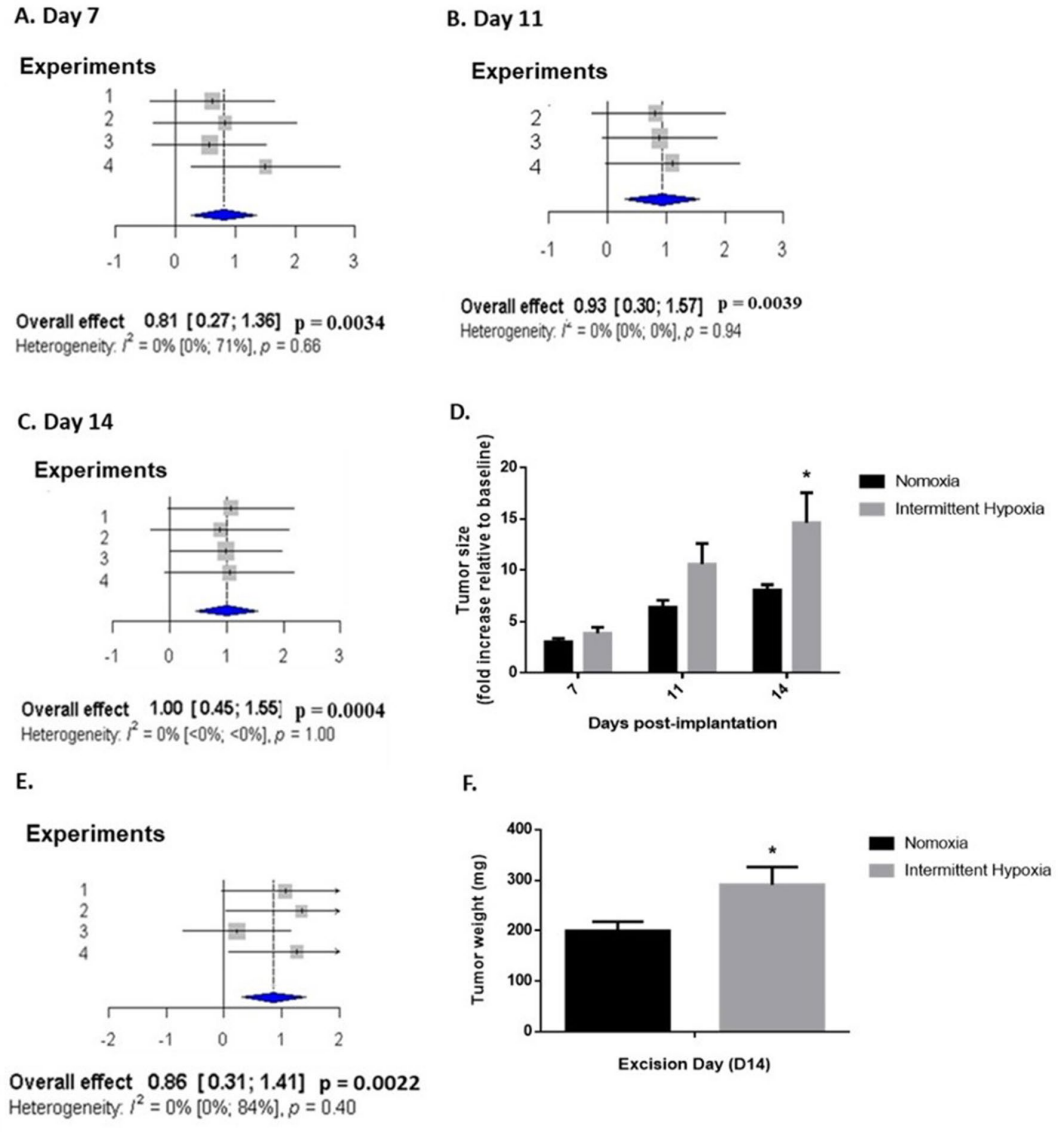
In vivo intermittent hypoxia exposure enhances breast tumor development and weight. (**A**, **B**, **C**, **E**) Forest plots presenting weighted standard mean differences in the effects of normoxia (N) and intermittent hypoxia (IH) exposure on tumor size at days 7 (**A**), 11 (**B**) and 14 (**C**) as well as tumor weight at day 14 (**E**) following orthotopic mammary implantation. Based on four independent experiments (n = 57 mice overall including 28 N and 29 IH mice). (**A**–**C**) Tumor growth, estimated by caliper measurement, was significantly increased at days 7, 11 and 14 (p = 0.0034, p = 0.0039 and p = 0.0004, respectively) of IH exposure. (**D**) Representative results from experiment 3 (n = 9 N and 9 IH mice) showing the kinetics of orthotopic 4T1 breast tumor growth (expressed relative to baseline value measured 3 days following implantation). *p < 0.05, repeated measures ANOVA. Data are presented as mean ± SEM. (**E**) Tumor weight was significantly increased (p = 0.0022) upon resection on day 14. (**F**) Representative results from experiment 2 (n = 6 N and 7 IH mice) showing 4T1 breast tumor weight after 14 days of N or IH exposure. *p < 0.05, student’s t-test. Data are presented as mean ± SEM.

In our experimental conditions, none of mice investigated died from IH exposure or cancer. Intermittent hypoxia induced a significant acceleration of tumor growth compared to normoxia (Fig. 4). Overall pooled effects size of 4 independent experiments, estimated by standard mean differences with Hedges’ method, was in favor of a significantly large effect of IH on tumor volume on day 7 (effect size = 0.81; [0.27–1.36]; p < 0.01) (Fig. 4A), day 11 (effect size = 0.93; [0.30–1.57]; p < 0.01) (Fig. 4B) and day 14 (effect size = 1.0; [0.45–1.55]; p < 0.001) (Fig. 4C) of exposure. In accordance, overall pooled effects size also highlighted a large effect of IH on tumor weight (effect size = 0.86; [0.31–1.41]; p < 0.01) on day 14 (Fig. 4E).

**Figure 5:**
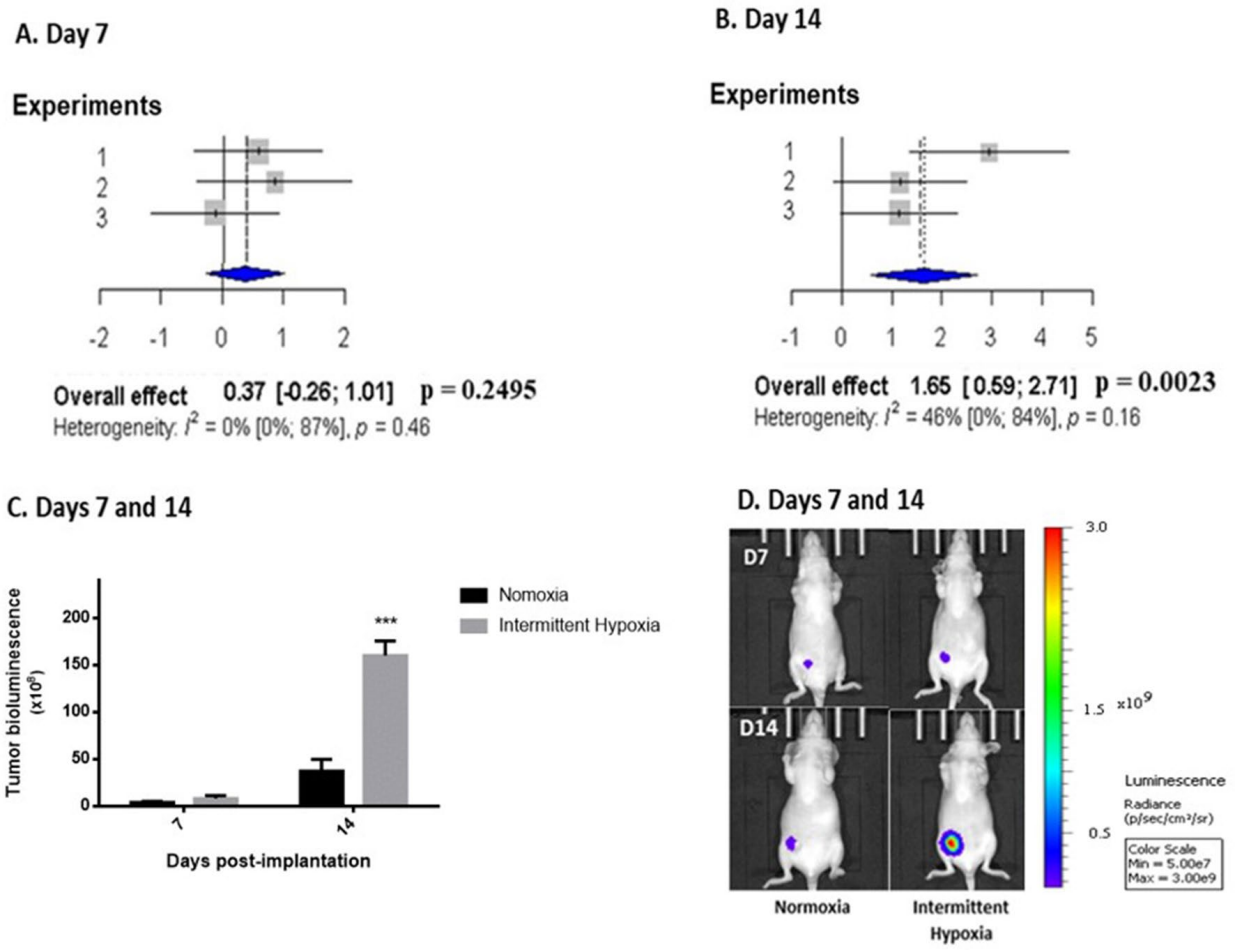
In vivo intermittent hypoxia exposure increases breast tumor cell proliferation estimated by bioluminescence imaging. (**A**, **B**) Forest plots presenting weighted standard mean differences in the effects of normoxia (N) or intermittent hypoxia (IH) on tumor growth, estimated by in vivo bioluminescence imaging at days 7 and 14 of exposure. Based on three independent experiments (39 mice overall including 19 mice exposed to N and 20 mice exposed to IH). Tumor growth on day 14 (**B**) was significantly increased (p = 0.0023) by IH exposure. (**C**) Representative results from experiment 1 (n = 6 N and 7 IH mice) showing enhanced tumor bioluminescence after 14 days of IH exposure. ***p < 0.001, repeated measures ANOVA. Data are presented as mean ± SEM. (**D**) Representative bioluminescence images showing the enhancing effect of intermittent hypoxia exposure 7 and 14 days after concomitant orthotopic implantation.

Finally, tumor cell proliferation, evaluated by in vivo bioluminescence imaging, was also significantly enhanced by 14 days of IH exposure compared to normoxia (Fig. 5). Overall pooled effects size of 3 independent experiments did not indicate a significant effect of IH on tumor growth on day 7 (Fig. 5A) but showed a large effect (effect size = 1.65; [0.59–2.71]; p < 0.01) on day 14 of exposure (Fig. 5B).

Individual data from the various experiments on tumor size, weight and bioluminescence imaging are presented in Supplemental Figs. S1–S3, respectively.

### Breast tumor expansion by in vivo intermittent hypoxia exposure is reduced by non-selective endothelin receptor antagonism

**Figure 6:**
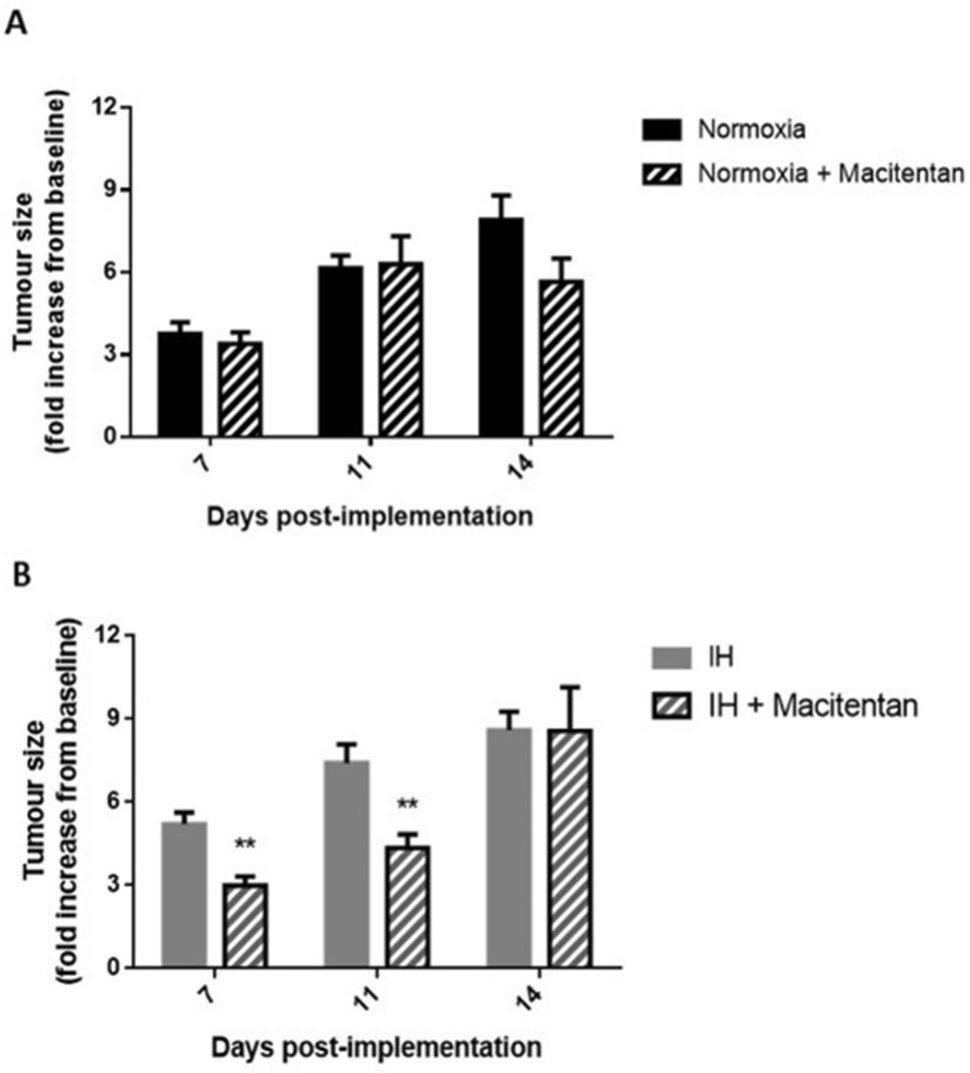
Non-selective endothelin receptor blockade reduces the tumor promoting effect of in vivo intermittent hypoxia exposure. The effects of daily oral administration of macitentan (30 mg/kg) on 4T1 orthotopic breast tumor volume (expressed relative to baseline value measured 3 days following implantation) were evaluated by caliper measurements on days 7, 11 and 14 of normoxia (N) or intermittent hypoxia (IH) exposure. Experiments were carried out on 4 groups (Normoxia, Normoxia + macitentan, IH, IH + macitentan) of mice (n = 7/group). (**A**) Macitentan treatment throughout normoxic exposure did not have significant effects (at day 14 p = 0.079 for N vs N + macitentan). (**B**) In contrast, macitentan treatment significantly reduced tumor size on days 7 and 11 of IH exposure. **p < 0.01, post-hoc analysis.

Oral macitentan administration throughout exposure did not affect tumor development in response to normoxia (Fig. 6A). In contrast, macitentan treatment significantly diminished tumor size on days 7 and 11 in animals exposed to IH (by 42.7% and 41.2%, respectively; p < 0.01) compared to untreated animals (Fig. 6B).

### In vivo intermittent hypoxia exposure promotes breast cancer metastasis

**Figure 7:**
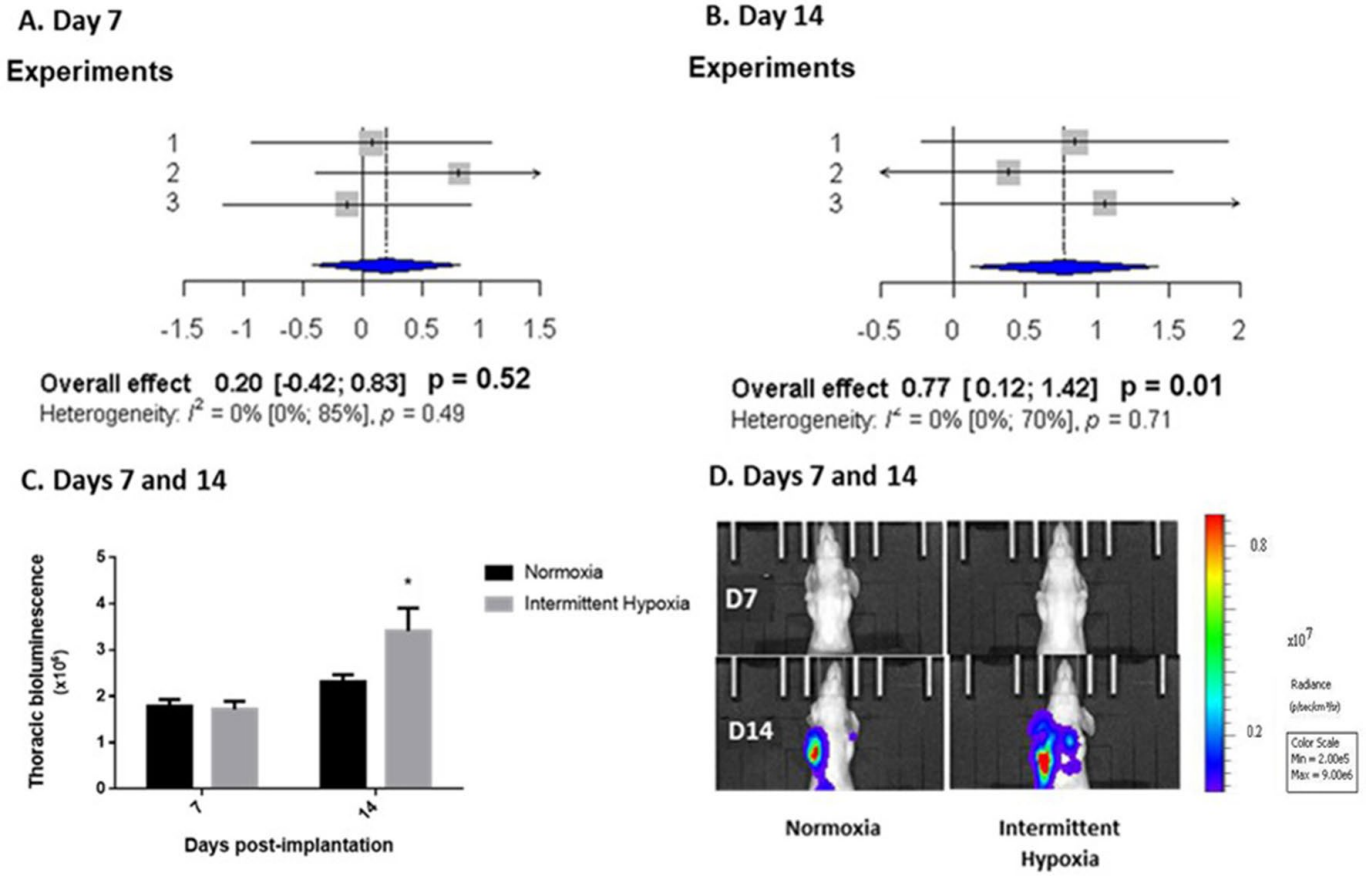
In vivo intermittent hypoxia exposure increases metastatic thoracic invasion estimated by bioluminescence imaging. (**A**, **B**) Forest plots presenting weighted standard mean differences in the effects of normoxia (N) and intermittent hypoxia (IH) on thoracic invasion, estimated by bioluminescence imaging, at days 7 (**A**) and 14 (**B**) of exposure. Based on three independent experiments (39 mice overall including 19 mice exposed to N and 20 mice exposed to IH). Thoracic metastatic invasion was significantly increased by IH exposure at day 14 (p = 0.01). (**C**) Representative results from Experiment 3 showing that exposure to intermittent hypoxia increased thoracic bioluminescence at day 14 (*p = 0.05, post-hoc analysis). Data are presented as mean ± SEM. (**D**) Representative bioluminescence images showing the enhancing effect of IH exposure 7 and 14 days after concomitant orthotopic implantation.

Metastatic thoracic invasion, estimated by in vivo bioluminescence imaging, was enhanced in mice exposed to 14 days of IH compared to normoxia (Fig. 7). Overall pooled effects size of 3 independent experiments did not indicate a significant effect of IH exposure on tumor growth on day 7 (Fig. 7A) but showed a moderate effect (effect size = 0.77; [0.12–1.42]; p = 0.01) on day 14 (Fig. 7B). Individual data from the various experiments are presented in Fig. S4.

Nevertheless, the proportion of animals with thoracic invasion was not statistically different in groups exposed to IH (80% and 100% at days 7 and 14, respectively) and normoxia (65% and 90% at days 7 and 14, respectively) and overall pooled effects size with Hedges’ method did not highlight significant differences (data not shown).

## Discussion

To the best of our knowledge, this is the first study to combine complementary in vitro and in vivo approaches to explore the effects of IH exposure mimicking OSA on breast cancer development and metastatic dissemination. Using devices specifically designed to mimic the IH pattern of OSA and a meta-analysis of at least three independent in vivo studies, we demonstrated that intermittent hypoxia enhances tumor growth and lung metastasis by promoting cell proliferation and migration, as demonstrated by in vitro experiments. Furthermore, we also showed that the deleterious effects of IH on breast cancer development could be reversed by blocking the endothelin pathway with the dual endothelin receptor antagonist macitentan.

### Intermittent hypoxia exposure enhances breast cancer growth

The effects of IH on primary breast tumor growth was evaluated by three methods: tumor volume measured by electronic caliper, tumor cell proliferation estimated by in vivo bioluminescence imaging and ex vivo tumor weight. Regardless of the method used, we observed that mice exposed to IH presented a significant increase in tumor growth, with a maximum effect on day 14 following mammary orthotopic implantation. Caliper measurements and bioluminescence imaging enabled us to show that IH exposure increased the kinetics of breast tumor development, and this was confirmed by an increase in ex vivo tumor weight.

These results are consistent with previously published in vivo data in melanoma^29,35,36^ or lung cancer^37,38^ models using slightly different IH protocols and mostly using ex vivo tumor weight as an endpoint.

Moreover, the results of the present study are strengthened by in vitro data showing that proliferation, migration and expansion were higher in tumor cells submitted to IH compared to normoxic exposure. This provides the first demonstration that in vivo breast tumor expansion by IH could be explained by both enhanced proliferation and increased migration capacity.

### Intermittent hypoxia exposure increases lung breast cancer metastasis

Thoracic metastasis, assessed by in vivo bioluminescence, was enhanced in mice exposed to IH. This is in accordance with our in vitro results showing increased migration and proliferation of breast cancer cells exposed to IH as shown by wound healing, transwell and proliferation assays. This is consistent with the in vitro study of Chen et al. showing that IH exposure increases the expression of genes known to predict lung metastasis in breast cancer^39^. Our results are also in accordance with the in vivo studies of Almendros et al. and Li et al. showing an enhanced lung metastasis of melanoma in rodents exposed to IH^29,31^.

### Inhibition of the endothelin pathway reverses the effects of intermittent hypoxia on tumor development

In view of the IH-induced increase of endothelin-1 secretion by 4T1 cells, we used macitentan, a dual endothelin receptor antagonist with enhanced tissue penetration and receptor binding properties and superior oral efficacy in animal models^40,41^, as a pharmacological tool to assess the role of ET-1 in the initiation and development of cancer in response to intermittent hypoxia. Macitentan treatment was able to reverse the in vitro effect of IH on breast cancer cell proliferation and migration and the in vivo cancer promoting role of the endothelin system was confirmed by a significant decrease in breast tumor size in IH-exposed mice treated with macitentan. These results are in line with our mechanistic hypothesis involving the ET-1 pathway in the deleterious effect of IH regarding tumor growth. Indeed, ET-1 overexpression has been shown in several tumors including breast cancer^19^, where it promotes tumor development through autocrine and paracrine effects on tumor cell proliferation, migration, and neovascularization^25^.

Since tumors are reported to be hypoxic, even in normoxic animals, one could have expected endothelin-1 secretion and thus macitentan effect on tumor growth in normoxic animals. Although macitentan treatment slightly tended to inhibit tumor growth after 14 days, its effect in mice exposed to normoxia was not statistically significant. In contrast, ET-1 receptor blockade was significantly effective in the context of IH exposure. This is in accordance with previous data from our group showing that while ET-1 receptor antagonism is devoid of effects in rodents exposed to normoxia, it has the ability to prevent the deleterious effects of IH on various organs such as the heart^17^, blood vessels^19^ and adipose tissue^21^. Moreover, in the present study, we investigated small, early-stage and thus presumably very mildly hypoxic tumors with presumably low intrinsic ET-1 expression. The significant effect of ET-1 receptor blockade in animals exposed to IH could thus be due to a specific effect of IH on endothelin expression in tumors, but also to the systemic nature of IH, which increases circulating ET-1 levels^18^ as well as tissue ET-1 levels in various organs, such as vessels^19^ and adipose tissue^21^, present in the tumor macroenvironment. This emphasizes the importance of investigating the contribution of healthy peripheral tissues to tumor development under IH conditions, in particular at the early stages of the disease. This issue was indeed highlighted by Hunyor et al. in a review mentioning the difficulty to distinguish between the contribution of the systemic IH associated with OSA from that of the local chronic hypoxia related to tumor growth^42^. Additional studies are required to explore the dynamic relationship between endothelin expression and oxygen levels in tumor, microenvironment and healthy tissues in order to assess their respective contribution to breast cancer expansion in the context of OSA-related systemic intermittent hypoxia.

Finally, additional studies with other cell types, such as other breast cancer cell lines but also cell lines from other types of cancers, will be required to confirm and establish the implication of ET-1 in IH-induced tumor growth.

## Conclusion

In summary, the present study is the first to show, using both in vitro and in vivo approaches, that chronic intermittent hypoxia exposure, similar to that encountered in obstructive sleep apnea patients, promotes breast cancer development and metastasis through activation of cell proliferation and migration. Moreover, prevention of the in vitro and in vivo effects of IH by macitentan is in favor of a major involvement of the endothelin system in the cancer-promoting effects of IH.

Our findings are of major importance to identify new risk factors and therapeutic tools that can be actionable for reducing the burden of cancer. There is current limited evidence to demonstrate a definite role of sleep apnea and intermittent hypoxia in cancer occurrence and progression. Available human studies are flawed by their retrospective design and no robust assessment of important confounders^43^. Our findings assembling complementary in vitro and in vivo approaches strongly support the concept that chronic intermittent hypoxia specifically activates pathophysiologic pathways, such as the endothelin system, underlying the link between sleep apnea and cancer. We are still in an early phase of the knowledge acquisition process and future investigations are warranted to better understand the clinical implications as well as the impact of endothelin receptor antagonism as a potential therapeutic approach for OSA patients with breast cancer.

## Supplementary Information

Supplementary Information.

## Data Availability

The datasets used and/or analysed during the current study are available from the corresponding author on reasonable request.

## Abbreviations

ET-1: Endothelin-1
HIF-1: Hypoxia-inducible factor 1
IH: Intermittent hypoxia
OSA: Obstructive sleep apnea
