# Supplementary material for: Chronic intermittent hypoxia, a hallmark of obstructive sleep apnea, promotes 4T1 breast cancer development through endothelin-1 receptors
Source: Sci Rep. 2022 Jul 28;12:12916. doi: 10.1038/s41598-022-15541-8 (PMC9334573; doi:10.1038/s41598-022-15541-8)
Supplement: Supplementary file 1 — Supplementary Information. [file 41598_2022_15541_MOESM1_ESM.docx]

**SUPPLEMENTARY MATERIAL**

Chronic intermittent hypoxia, a hallmark of obstructive sleep apnea, promotes 4T1 breast cancer development through endothelin-1 receptors

Mélanie Minovés^1*^, Sylvain Kotzki^1^, Florence Hazane-Puch^2^, Emeline Lemarié^1^, Sophie Bouyon^1^, Julien Vollaire^3^, Brigitte Gonthier^1^, Jean-Louis Pépin^1^, Véronique Josserand^3**^, Anne Briançon-Marjollet^1**^, Diane Godin-Ribuot^1**^

*1 Univ. Grenoble Alpes, Inserm, CHU Grenoble Alpes, HP2, 38000 Grenoble, France*

*2 CHU Grenoble Alpes, CS 10217, 38000, Grenoble, France*

*3 Univ. Grenoble Alpes, CNRS, Inserm, CHU Grenoble Alpes, IAB, 38000 Grenoble, France*

** Co-last authors

* Corresponding author:

Mélanie Minovés

Laboratoire HP2, Inserm U1030, Université Grenoble Alpes

UFR de Pharmacie de Grenoble, 38700 Grenoble, France

e-mail address: [mminoves@chu-grenoble.fr](mailto:mminoves@chu-grenoble.fr)

**Statistical method for meta-analysis**

We opted for meta-analysis in order to avoid having to combine the results of independent studies or to retain only certain results. Meta-analyses of tumor size, assessed on days 7, 11 and 14 after tumor cell implantation, and tumor weight, assessed on day 14, were performed from four independent experiments. Tumor growth and thoracic dissemination, assessed by *in vivo* bioluminescence imaging on days 7 and 14 after tumor cell implantation, were performed from three independent experiments.

Continuous outcome data were analyzed with *metacont* function from the *meta* package (version 4.13-0; RStudio version 1.2.1335, Boston, USA), using standardized mean difference (SMD) to express effect size in each experiment relative to the observed variability:

$$SMD=\frac{\left( Mc-Me \right)}{SD}$$

where Mc is the mean outcome measure in the control group, Me is the mean outcome measure in the experimental groups, and SD is the pooled standard deviation of the two groups. We used the Hedges’ method to estimate the summary measure *g (overall effect)*.

When data were expressed as effective (number of mice with positive thoracic invasion in IH- and normoxia-exposed groups) we used the *escalc* function from the *metafor* package (version 2.4-0), using the (log) relative risk as outcome measure of effect size in each experiment.

Homogeneous data were analyzed using fixed-effect models and heterogenous data were analyzed using random effect size models. We considered data as heterogeneous when the I² value was superior to 50%.

***
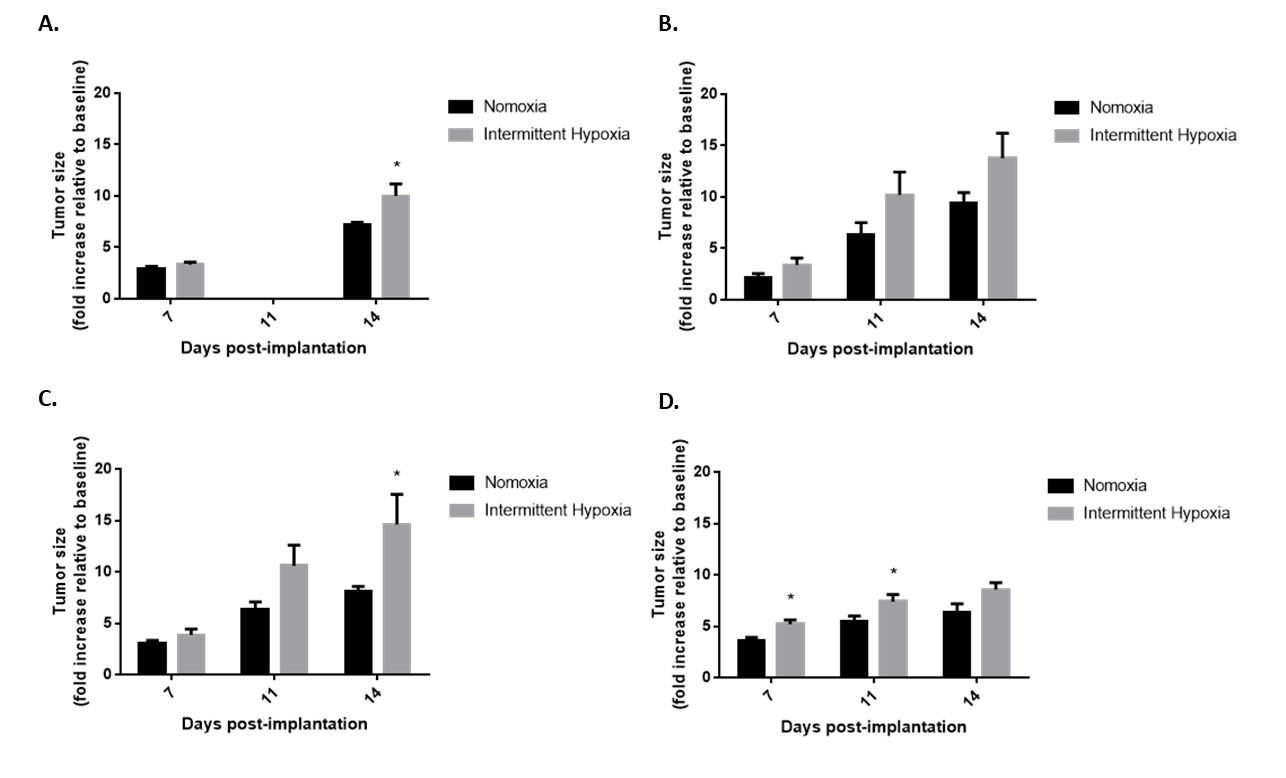
***

**Figure S1. Effects of normoxia and intermittent hypoxia exposure on orthotopic breast tumor growth in 4 independent experiments.**

Kinetics of 4T1 breast tumor growth were evaluated on days 7, 11 and 14 of exposure to normoxia (N) or intermittent hypoxia (IH) immediately after orthotopic implantation. Tumor size was assessed by repeated caliper measurements and expressed relative to baseline value (measured 3 days following implantation).

The four experiments were performed on (**A**) 13 (6 N and 7 IH), (**B**) 12 (6 N and 6 IH), (**C**) 18 (9 N and 9 IH) and (**D**) 14 (7 N and 7 IH) mice, respectively. Significant effects of IH exposure were observed on day 14 in two experiments (*p<0.05, **A** and **C**) and on days 7 and 11 in one experiment (*p< 0.05, **D**). Data are presented as mean ± SEM.


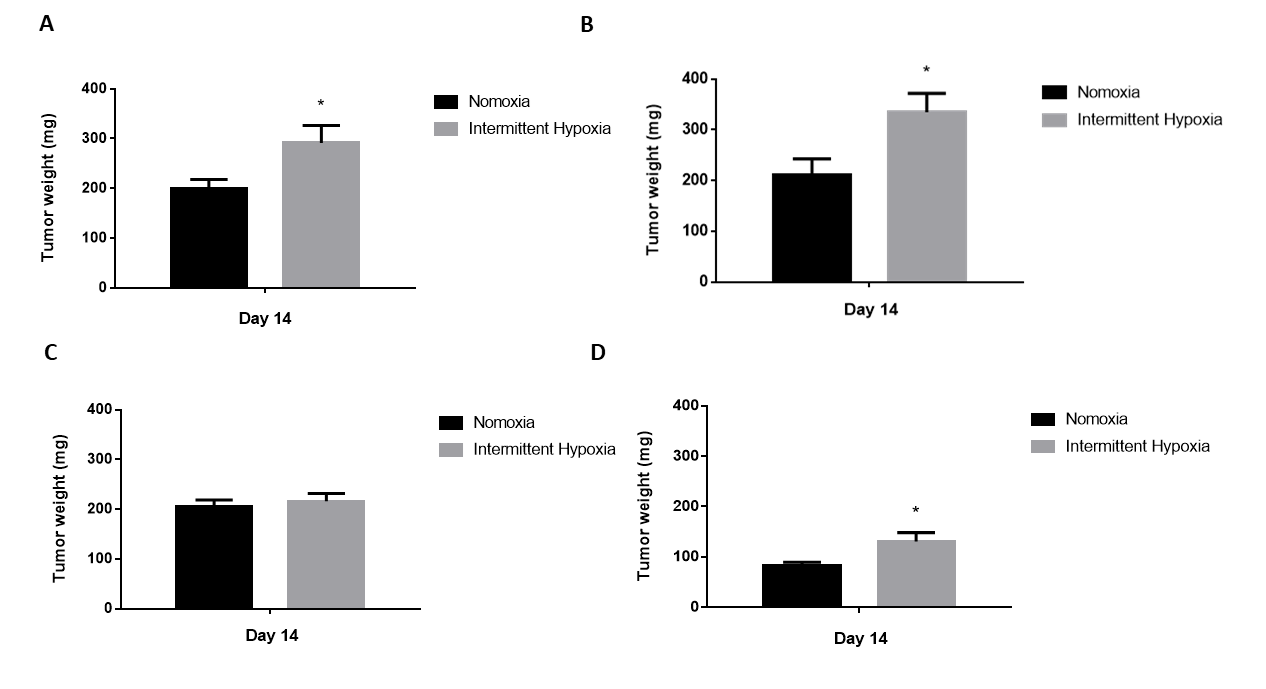


**Figure S2. Effects of normoxia and intermittent hypoxia exposure on orthotopic breast tumor weight in 4 independent experiments.**

4T1 breast tumors were excised and weighted on day 14 of normoxia (N) or intermittent hypoxia (IH) exposure. The four experiments were performed on (**A**) 13 (6 N and 7 IH), (**B**) 12 (6 N and 6 IH), (**C**) 18 (9 N and 9 IH) and (**D**) 14 (7 N and 7 IH) mice, respectively. Significant effects of IH exposure were observed in three experiments (*p<0.05, **A**, **B** and **D**). Data are presented as mean ± SEM.

***
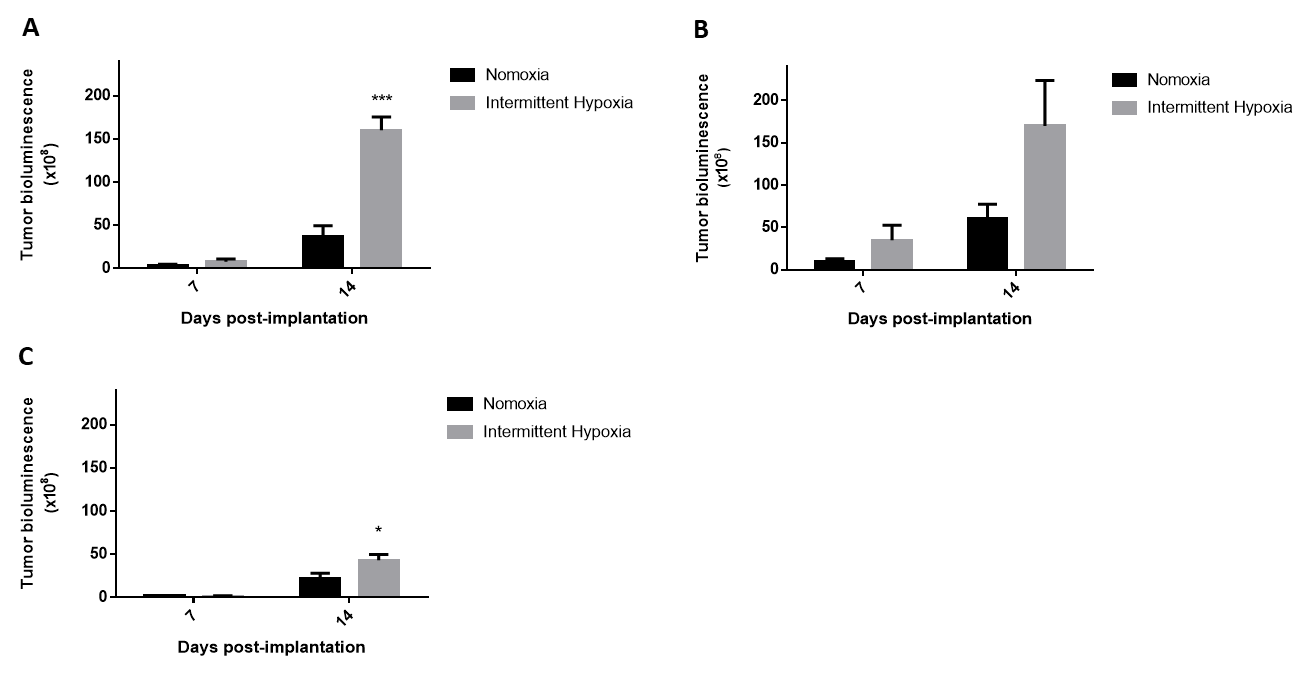
***

**Figure S3. Effects of normoxia and intermittent hypoxia exposure on tumor growth estimated by *in vivo* bioluminescence imaging in 3 independent experiments.**

4T1 breast tumor growth was estimated on days 7 and 14 of normoxia (N) or intermittent hypoxia (IH) exposure. The three experiments were carried out on (**A**) 13 (6 N and 7 IH), (**B**) 12 (6 N and 6 IH) and (**C**) 14 (7 N and 7 IH) mice, respectively. The bioluminescence signal was significantly increased on day 14 in two experiments ( ***p < 0.001, *p < 0.05, **A** and **C**) and a strong tendency was seen in the third one (p=0.064, **B**). Data are presented as mean ± SEM.

***
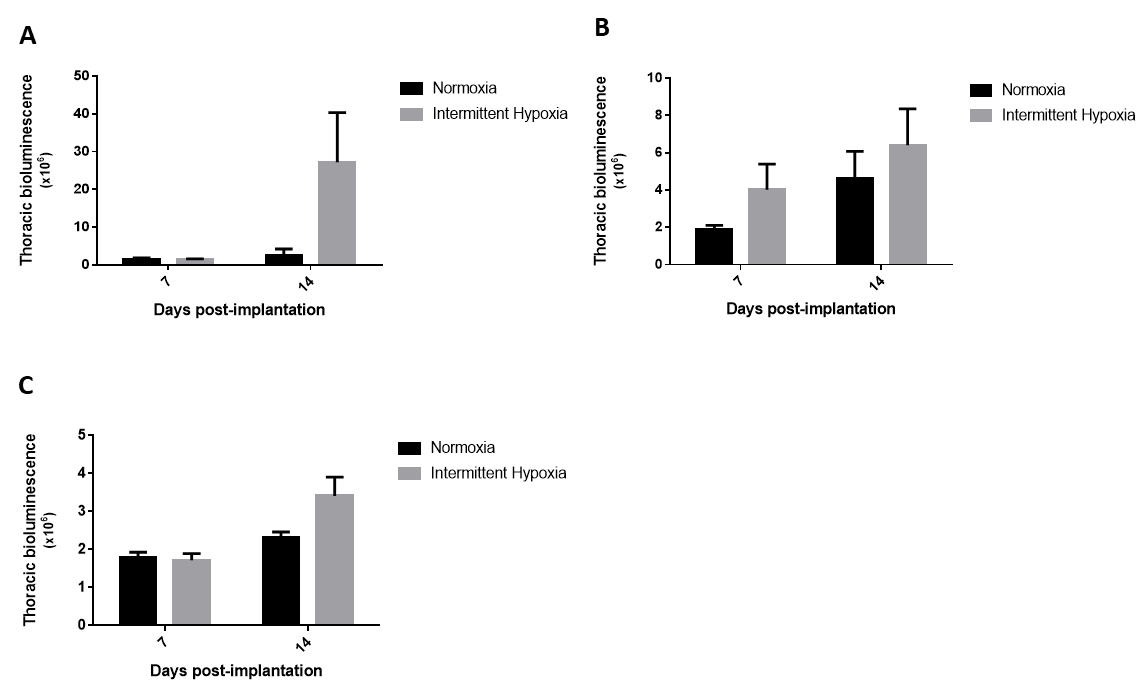
***

**Figure S4. Effect of normoxia or intermittent hypoxia exposure on thoracic invasion estimated by bioluminescence.**

Thoracic cancer cell invasion, estimated by bioluminescence imaging, was assessed in three independent experiments on (**A**) 13 (6 N vs 7 HI), (**B**) 12 (6 N vs 6 HI) and (**C**) 14 (7 N vs 7 HI) mice, respectively.

No significant difference between the effects of normoxia and intermittent hypoxia exposure was observed at all time points, although a strong tendency was observed at day 14 in the third experiment (p=0.055, **C**). Data are presented as mean ± SEM.
